# Genetic Variability and Population Structure of the Tunisian Sicilo-Sarde Dairy Sheep Breed Inferred from Microsatellites Analysis

**DOI:** 10.3390/genes13020304

**Published:** 2022-02-05

**Authors:** Yousra Ben Sassi-Zaidy, Aziza Mohamed-Brahmi, Ghada Nouairia, Faouzia Charfi-Cheikhrouha, M’Naouer Djemali, Martino Cassandro

**Affiliations:** 1 Laboratory of Diversity, Management and Conservation of Biological Systems, LR18ES06, Faculty of Sciences of Tunis, University of Tunis El Manar, El Manar II Tunis 2092, Tunisia; facharfi@yahoo.fr; 2 Department of Agronomy, Animal, Food, Natural Resources and Environment, University of Padova, 35020 Legnaro Padova, Italy; ghada.nouairia@dbb.su.se; 3 Laboratory of Animal Genetic and Feed Resources Research, Department of Animal Science, Institut National Agronomique de Tunis, University of Carthage, Tunis-Mahrajène Tunis 1082, Tunisia; mdjemali@webmails.com; 4 Laboratory of Agricultural Production Systems Sustainability in the North Western Region of Tunisia, Department of Animal Production, Ecole Supérieure d’Agriculture du Kef Boulifa, University of Jendouba, Le Kef 7119, Tunisia; mohamedaziza2003@yahoo.fr; 5 Department of Biochemistry and Biophysics, Stockholm University, SE-10691 Stockholm, Sweden

**Keywords:** genetic diversity, population structure, Sicilo-Sarde dairy sheep, microsatellite markers, conservation

## Abstract

This study analyzed the genetic variability, inbreeding and population structure of the Tunisian–North African dairy sheep breed, the Sicilo-Sarde (SS), created by crossing the Sarda and Comisana dairy breeds. The level of variability in the SS, considered as an endangered breed after a dramatic decrease, was assessed using 17 microsatellite markers by analyzing the two breed populations sampled from their respective cradles: SS of Beja (SS_B_, *n* = 27) and SS of Mateur (SS_M_, *n* = 25). High levels of genetic diversity in SS were revealed, with a total of 212 alleles, a high mean number of alleles (12.47 ± 4.17) and a high average polymorphism information content (PIC) (0.81 ± 0.10). The observed heterozygosity was considerable in SS_B_ and SS_M_ (0.795 and 0.785, respectively). The inbreeding level measured by the population inbreeding coefficient *F_IS_* is higher in the SS_M_ population (0.121) than in the SS_B_ population (0.090). The higher genetic diversity level detected in SS_B_ reflected the effect of new Italian Sarda genes introduced by intra-uterine artificial insemination recently practiced in this population. The Wilcoxon test and the mode-shift distribution indicated that the SS breed is a non-bottlenecked population. The structural analysis reflected the historical miscegenation practiced during the breed creation and highlighted further ancient miscegenation, which could date back to the first waves of sheep introduction to the western Mediterranean region. Microsatellite markers were successfully applied in the assessment of the genetic variability of SS and should be used in monitoring this variability during the application of conservation strategies.

## 1. Introduction

**Figure 1:**
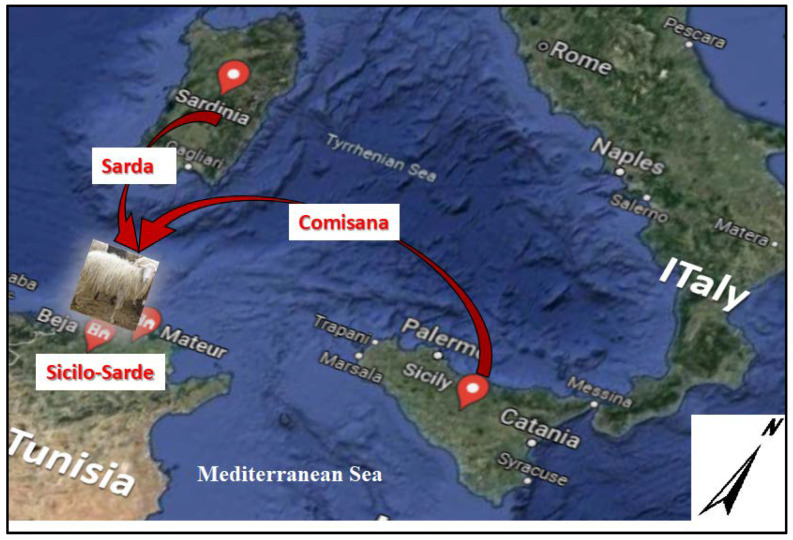
Locations of the breeding area of the Sicilo-Sarde dairy breed and its ancestors Comisana and Sarda.

Milk became available for human consumption, since the onset of the Neolithic, between 12,000 and 9000 years ago in the Fertile Crescent identified as a domestication center [1,2]. Following terrestrial and Mediterranean roads, the Neolithic culture characterized by sheep and goat milk consumption become widespread and reached the west Mediterranean basin in the seventh millennium [3,4]. Since that time and until today, dairy sheep farming has been concentrated in the Mediterranean basin [4,5,6,7]. Nowadays, this region encloses 41.4% of dairy ewes, producing approximately 41.4% of the entire world’s sheep milk [7]. Currently, Southern Europe has many specialized dairy sheep breeds. Nevertheless, in the southern Mediterranean rive, it was only at the beginning of the 17th century when the unique dairy sheep breed in Tunisia and North Africa was created by the Italian community, specifically in Tunisia, to meet their cheese consumption needs [8]. This dairy sheep breed, called Sicilo-Sarde, was developed in Northern Tunisia by a long-term crossing between two Italian dairy sheep breeds: Sarda originated from Sardinia and Comisana from Sicilia [8], hence the name Sicilo-Sarde. Since the creation of this breed in the northern sub-humid region of Tunisia, specifically in the Beja and Bizerte regions, dairy sheep farms remain on the hills and in the plains of northern Tunisia (Figure 1), with a semi-extensive breeding system still used today, with similar climatic conditions to that of most dairy sheep farms in Sardinia [7]. However, this dairy breed has shown its adaptability to the warmer and dried Tunisian summer.

The ability of the SS to produce milk makes it the only dairy breed in the North African region despite its quite limited production potential obtained after a long suckling period practiced by breeders [6]. This specificity requires dual-purpose breed management (milk and meat) for this originally specialized dairy sheep [6]. In fact, the average milk production of this breed, in 90% of ewes, is more than 80 L and can reach 117 L/ewe/lactation using an early weaning of 42 days, with an undefined total milking period [8,9]. This production remains comparable with that of Italian ewes, with an average of 84.6 L [7]. The productivity of the two breeds’ ancestors Sarda and Comisana reached 201 and 159 L/ewe/lactation, respectively [7]. Although this truncated quantitative production is explained by this breeding strategy, the milk quality of the SS and its cheese are classified within the high range of Mediterranean dairy breeds [9,10]. Its milk fat, protein and casein contents, on which cheese yield depends [9], were 7.49%, 6.55% and 5.16%, respectively [8]. In a more recent study [9], these parameters reached 6.85%, 5.93% and 4.74%, respectively. Pulina et al. [7] in a 5-year survey of Italian Sarda milk designed to produce the Pecorino Romano cheese, considered the first sheep cheese at an international level in terms of quality and values, recorded 6.48%, 5.59% and 4.31% milk fat, protein and casein percentages, respectively. Rouissi et al. [8] revealed the superiority of SS in terms of protein and casein contents than Comisana reared in a same Tunisian farm conditions (6.56% and 5.16% for SS and 6.4% and 4.97% for Comisana, respectively). In the Italian rearing conditions, Comisana sheep recorded 7.31% fat and 6.14% protein [11]. Liotta et al. [12] revealed a fat percentage varying from 5.58% to 6.68%, a protein percentage from 4.92% to 6.09% and a casein percentage from 4.85% to 5.81% in Italian Comisana sheep reared in Sicily. All of these cited results highlighted the excellent milk quality of the SS sheep. Despite this high quality, SS milk is limited to micro-sector cheese production mainly characterized by handmade quality yogurts and cheeses, especially the “Sicilian” cheese with a uniquely “terroir” reputation [10,13].

The sustainability of the sheep-milk cheese production in Tunisia has been threatened in recent decades after a dramatic decrease in the number of dairy breed (90% of the total number). Its decline from 200,000 female units in 1991 to 26,500 units females in 1995 and to 25,000 in 2000 indicate that this breed faces a threat of extinction and that the situation has reached a critical level [8,10]. The low prices of sheep milk as well as the shift to the breeding of dairy cattle were among the main causes of this decline. Faced by these changes, many alternatives were undertaken by national and international institutions to save this breed. In 2003, the creation of the Sicilo-Sarde Breed Association by private farmers in the Beja region reversed the critical situation of the breed into a winning one following an efficient triple interaction between farmers, researchers and policy makers [6,10]. Under a multidisciplinary improvement program, new genes from the ancestral Italian Sarda breed were introduced by intra-uterine artificial insemination to the Tunisian dairy sheep population of Beja.

Since the climate change is expected to make overall climates warmer and dryer, the hardy indigenous sheep breeds that have proven their adaptability to increasing temperatures are considered a main way to meet not only international food demands but also environmental sustainability [14]. These sheep’s genetic resources should be identified and characterized using genomic tools to be preserved for future production systems [15]. Therefore, the objective of this study was to characterize the unique native dairy sheep breed in Tunisia and North Africa using an informative set of microsatellite markers by analyzing the two populations of this sheep: the population of Beja and the population of Mateur. This work assesses (1) the two populations’ levels of genetic variability, (2) the level of risk due to declining enrollment in this breed and the detection of a bottleneck effect, and (3) the efficiency of improvements in the breed’s genes in Beja.

## 2. Materials and Methods

### 2.1. Sampling Design and Genotyping

A total of 52 individuals were sampled from two populations of the dairy Sicilo-Sarde native breed: SS_B_ from Beja region (*n* = 27) and SS_M_ from Mateur region (*n* = 25). Unrelated animals were sampled from each farm or small flock based on the information provided by farmers and breeders when pedigree data were not available. The out-group sheep breeds, from Tunisia (Barbarine (BAR, *n* = 30), Queue Fine de l’Ouest (QFO, *n* = 30), Noire de Thibar (NTH, *n* = 30)); from Morocco (D’man (DM, *n* = 28)); and from Italy (Appenninica (APP, *n* = 31) and Lamon (LAM, *n* = 30)), were used to explore the population structure and the genetic uniqueness level of the SS. Genomic DNA extraction was carried out from 200 µL of whole blood using the Wizard Genomic DNA Extraction Kit (Promega, Madison, WI, USA) following the manufacturer’s protocol. The total of samples were genotyped at 17 microsatellites loci panel established from the ISAG/FAO recommended microsatellites markers [16] and from previous sheep genetic diversity studies [17,18,19,20,21]. The genotypes for all 17 microsatellite markers were determined by means of three multiplex PCR reactions using fluorescence-labeled primers in a total volume of 12.5 µL. Amplification was performed using standard PCR reactions in a GeneAmp 9700 thermal cycler (Life Technologies, Carlsbad, CA, USA) starting from 50 ng of purified DNA. The 17 microsatellites were amplified with the following conditions: initial denaturation step of 5 min at 95 °C, 35 cycles of 30 s at 95 °C, 1 min 30 s at 61 °C, 30 s at 72 °C and a final extension of 30 min at 60 °C. Multiplexes pooled by capillary electrophoresis and allele size were performed with a CEQ 8000 Genetic Analysis System (Beckman Coulter, Fullerton, CA, USA).

### 2.2. Statistical Analysis

The MICRO-CHECKER version 2.2.3 [22] software was used for assessing genotyping quality, occurrence of null alleles, stuttering and allelic dropout. Exact tests for deviations from the Hardy–Weinberg Equilibrium (HWE) and linkage disequilibrium (LD) were applied using GENEPOP version 4.3 [23]. The number of alleles per locus (NA), mean number of alleles (MNA), allelic frequencies, observed (H_o_) and expected (H_e_) heterozygosity, and gene flow (Nm) were calculated using GENETIX version 4.05.2 [24]. The number of private alleles (*P*_AR_) in the different populations using the rarefaction method was counted using ADZE software [25]. The MSA software [26] was used to calculate allelic richness (AR, the mean number of alleles per locus corrected by sample size) and Wright’s fixation indices (*F*_IS_, *F*_IT_ and *F*_ST_; [27]). The Polymorphic Information Content (PIC) was measured using Molkin 3.0 [28]. Nei’s [29] genetic (D_A_) and *F*_ST_ pairwise distances among populations was estimated using GENETIX version 4.03 [24]. This software was likewise used to perform the factorial correspondence analysis (FCA) based on individual multilocus genotypes. As the SS breed has suffered from a dramatic population size reduction during recent decades, the BOTTLENECK software v.1.2.02 [30] was applied using two different tests: (1) the Wilcoxon test [31], to assess the bottleneck hypothesis under the three mutation models, infinite alleles model (IAM), stepwise mutation model (SMM) and two-phased mutation model (TPM), which examines the departure from mutation-drift equilibrium based on heterozygosity excess or deficiency (examines differences between the observed and the expected heterozygosity based on the observed number of alleles), and (2) the allele frequency distribution test, which is a graphical method comparing the frequencies of all alleles in the population to the distribution expected at mutation-drift equilibrium [31]. *F*_ST_ distances among breeds were represented by a neighbor-network graph constructed using Splits Tree4 software [32]. The genetic structure and population degree of admixture were investigated using the Bayesian clustering approach implemented in STRUCTURE software v.2.3.4 [33]. To choose the appropriate number of inferred clusters to model the data, 50 independent runs were performed for each cluster K (2 < K < 5). All analyses used a burn-in period of 30,000 and 150,000 iterations for data collection. The optimal number of clusters fitting the data was established following the Δ K method [34]. The output obtained was used directly as input data in the cluster visualization programs DISTRUCT [35] and CLUMPAK [36].

## 3. Results and Discussion

### 3.1. Genetic Diversity

All seventeen screened loci were included in the data, since no evidence of null alleles, stuttering bands or large allele dropout was found. A summary of the polymorphism of the genotyped markers is given in Table 1. The total number of alleles detected across the microsatellites markers was 212 different alleles. Except the locus MAF214, all microsatellite loci were found to be highly polymorphic, with PIC values larger than 0.70 and a high PIC average (0.81 ± 0.10), as shown in Table 1. The number of alleles per locus ranged from 5 to 18 (mean = 12.47 ± 4.17). The allelic richness considering all loci was equal to 9.55 ± 2.81 (Table 1). The fixation indices *F*_IS_, *F*_IT_ and *F*_ST_ for each locus and across all loci are given in Table 1. The mean values of these parameters were *F*_IS_ = 0.100 ± 0.110, *F*_IT_ = 0.110 ± 0.100, and the *F*_ST_ index was equal to 0.010 ± 0.020 (*p* < 0.001). The result of the genetic variability parameters revealed that the microsatellites panel used in this study was highly informative and appropriate for characterizing the unique Tunisian and North African dairy breed. The usefulness of this panel was endorsed by previous sheep genetic diversity studies [18,19,20,21]. Lower values of these parameters were detected in four Tunisian [37] and in Moroccan [38] and Algerian [39] sheep breeds.

Microsatellite markers still remain an appropriate tool for first-stage exploration of the genetic diversity and the population structure of sheep [40] and animal populations [41]. The allelic diversity of the two dairy breed populations SS_B_ and SS_M_ was studied in terms of MNA, AR and *P*_AR_, as summarized in Table 2. The MNA values calculated in the two populations SS_B_ and SS_M_ were similar to the values reported by [42] in the two ancestor Italian dairy breeds of SS: Comisana (9.47 ± 2.93) and Sarda (9.42 ± 2.89). Kdidi et al. [37] revealed a lower value of MNA (9.23) within the SS breed. After adopting the rarefaction method, the maximal AR (7.30 ± 1.94) was revealed in SS_M_ population (Table 2).

During the first investigation of Tunisian sheep by microsatellite markers, Ben Sassi-Zaidy et al. [19] found a higher AR (8.18) in the SS dairy breed; however, a similar value (7.27) was revealed by Kdidi et al. [37] in this breed. As mentioned by Tolone et al. [42], the ancestral breeds Comisana and Sarda exhibited higher AR (8.73 ± 2.71 and 8.39 ± 2.32, respectively). The *P*_AR_ values in the two populations of SS breed were quite similar (Table 2) as well as similar to the *P*_AR_ values revealed by Ben Sassi-Zaidy et al. [19] and by Kdidi et al. [37] in the SS breed. In general, the level of *P*_AR_ in the Tunisian dairy breed is similar to the level of this parameter in specialized breeds: 0.72 in D’man, the prolific sheep breed of North Africa [19]. In dairy cattle, Maretto and Cassandro [43] revealed an important *P*_AR_ (0.97) in highly selected Italian Holstein Friesian cattle, but small values (0.07 to 0.19) were recorded in the native Italian Burlina cattle breed.

For the two dairy populations, the average H_o_ across loci was considerable and slightly lower than H_e_ (Table 2). The deviation from the Hardy–Weinberg equilibrium (E) after Bonferroni correction (*p* < 0.05) was quite limited (just one locus: CSRD and TGLA in SS_B_ and SS_M_, respectively). The H_o_ values within the SS breed were similar to that observed in its ancestral breed the Italian Sarda (H_o_ = 0.71 ± 0.108; [42]) but higher than the value (0.644 ± 0.147) revealed by Kdidi et al. [37] in the Tunisian SS. The SS_M_ population belonging to the OTD public farm exhibited the highest inbreeding level (*F_IS_* = 0.121 ± 0.016) despite the limited deviation from the HWE. These results can be explained by the management strategy that is followed by the OTD office, which is based on traditional farming with a simple paternity control and without selection pressure reflecting the absence of selective forces acting on the studied loci or on the closely linked genes. Furthermore, this inbreeding level could have resulted from the dramatic reduction in sample size for the SS in the nineties. This fact seems to be slightly corrected in the population SS_B_ of Beja (*F*_IS_ = 0.090 ± 0.038) after the introduction of new Italian Sarda genes by intra-uterine artificial insemination, made available in 2004/2005 under the supervision of the Sicilo-Sarde Breed Association created in the Beja region. Tolone et al. [42] revealed that the higher values of inbreeding ranging from 0.314 to 0.645 were explained mainly by a genetic drift due to the selection pressure practiced on the microsatellites linked gene in Sicilian breeds including Sarda and Comisana. The very low *F*_ST_ index (0.0096) indicated a very low differentiation between the SS_B_ and SS_M_. In fact, only 0.96% of the genetic variability is explained by difference between the two sub-populations, and a large part of the total variability (99.04%) is explained by the difference among individuals. Thus, the heterozygosity deficiency revealed in the SS breed is explained by inbreeding rather than the Wahllund effect. Ben Sassi-Zaidy et al. [20] revealed a lower value of genetic variability among the Barbarine Tunisian sheep ecotypes (0.7%). A higher value (4.9%) of the total genetic diversity was explained by the differentiation among Sardinian and Sicilian sheep [42].

Signs of a strong reduction in the SS’s population size were assessed by analyzing the bottleneck hypothesis. An evaluation of the departure from the mutation-drift equilibrium based on heterozygosity excess for both populations SS_B_ and SS_M_, under the three mutation models IAM, TPM and SMM were conducted using the Wilcoxon test (Table 3).

The heterozygosity tests revealed a significant heterozygosity excess in the SS_B_ and SS_M_ populations under the IAM mutation model; in contrast, these two populations were shown at mutation-drift equilibrium under the SMM and TPM models. Since the TPM model is recommended for microsatellites analysis [30], no population displayed significant heterozygosity excess, suggesting a correction of the bottleneck effect. In fact, both the SS_B_ and SS_M_ populations showed normal L-shaped allele frequency distributions, indicating a non-bottlenecked population. This situation can probably be explained by the relatedness and the high gene flow between SS flocks, since the majority of SS_B_ individuals of Beja belonged to a large corporation before being distributed as small herds to many small breeders under the control of the Sicilo-Sarde Breed Association, thus maintaining sufficient diversity levels. Radha et al. [44], and Maretto and Cassandro [43], investigating small size sheep and cattle native breeds, respectively, using microsatellite markers, revealed the absence of bottleneck events despite the reduction in population size of these breeds.

**Figure 2:**
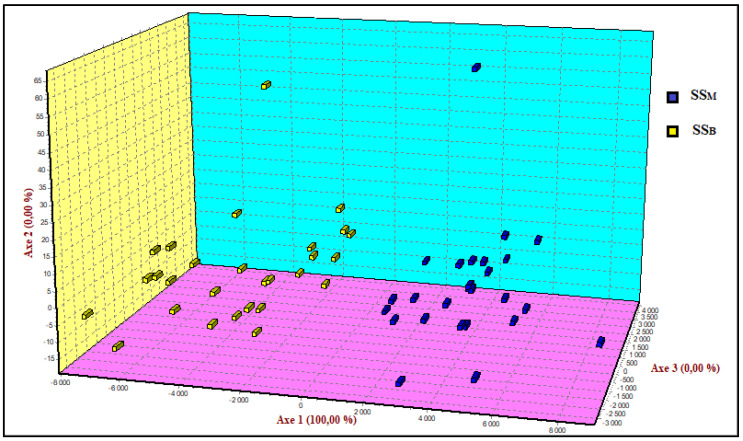
Spatial representation of the Tunisian dairy sheep breeds SS_B_, the Sicilo-Sarde population of the Beja region, and SS_M_, the Sicilo-Sarde population of the Mateur region, as defined by the factorial correspondence analysis based on all microsatellite loci and corresponding allele frequencies. The first three axes explained 100% of the total variation; the share of each axis is indicated by the value in parentheses.

To better understand the relatedness of the two populations of SS breed, we calculated the gene flow (Nm) and the Nei’s (DA) genetic distances between SS_B_ and SS_M_, which are 25.85 and 0.051, respectively. The important values of Nm and the low genetic distance between these two populations, despite the geographic distance between the regions Beja and Mateur, revealed their parental relatedness, since both were created by crossing the Italian Comisana and Sarda at the beginning of the last century. The factorial correspondence analysis was performed using the two populations and all loci with their corresponding allele frequencies (Figure 2). The total variation was minimized in only the first component (Axis 1), which separated individuals from the two populations SS_B_ and SS_M_. No separation was found between the two sheep populations following axis 2 and axis 3.

### 3.2. Genetic Structure and Breed Differentiation Analyses

#### 3.2.1. Genetic Structure Analysis

**Figure 3:**
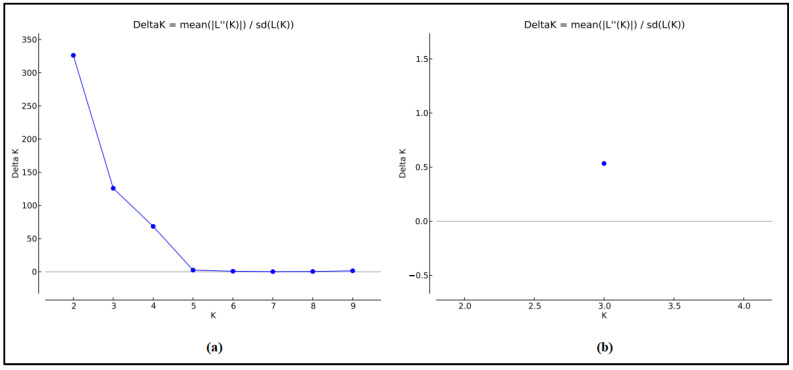
Optimal number of ancestral populations according to the ΔK method. (**a**) For all analyzed breeds, the best assumed cluster is K = 2. (**b**) For the two Sicilo-Sarde populations, the best assumed cluster is K = 3.

**Figure 4:**
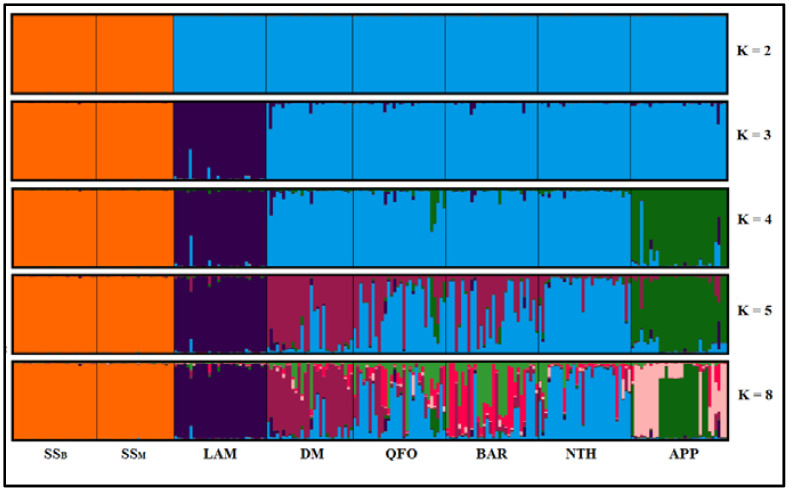
Estimated genetic structure of the unique North African dairy sheep, the Sicilo-Sarde, for assumed K ranging from K = 2 (best assumed K) to K = 8. SS_B_ = Sicilo-Sarde of Beja region; SS_M_ = Sicilo-Sarde of Mateur region; Tunisian out-group breeds: BAR = Barbarine; QFO = Queue Fine de l’Ouest; NTH = Noire de Thibar; DM = D’man; Italian out-group breeds: APP = Appenninica; LAM = Lamon.

The genetic structure of SS dairy breed was investigated with K number of expected clusters ranging from 1 to 9, using different out-group breeds from Tunisia—Queue Fine de l’Ouest (QFO), Noire de Thibar (NTH), Barbarine (BAR) and D’man (DM)—and from Italy—Appenninica (APP) from the Center of Italy and Lamon (LAM) from the Veneto region. The optimal number of ancestral populations according to the method of Evanno et al. [34] was K = 2 (Figure 3a). At K = 2, the uniqueness of the SS dairy breed, which was the first to be differentiated from the other Italian and Tunisian sheep breeds, is demonstrated in Figure 4. This result can be explained by the dairy specificity of the SS, since all the out-group breeds have meat vocation. Tolone et al. [42], assuming K = 2 expected clusters, revealed the distinctness of the Barbaresca breed originating from Barbarine North African meat sheep from the group formed by the dairy Sicilian and Sardinian sheep.

At K = 3 (Figure 4), the Venetian LAM breed was differentiated from the group formed by the Tunisian meat breeds (DM, QFO, BAR and NTH) and the Italian APP, which was separated at K = 4. At higher K values, the cluster including the two SS populations is still clearly distinguishable. Two other breed-specific clusters, the DM and the NTH clusters, appeared in the Tunisian meat sheep group. The BAR and the QFO Tunisian breeds showed an admixture-like pattern between the DM and the NTH clusters (Figure 4, K = 5).

**Figure 5:**
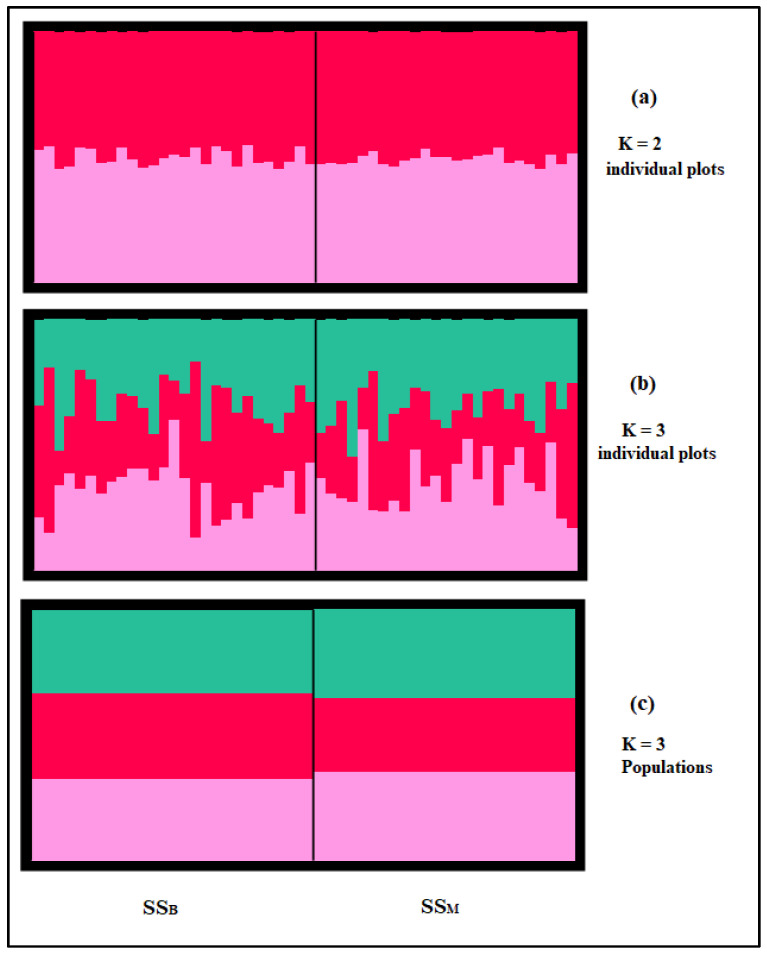
Estimated population structure of the Sicilo-Sarde dairy populations represented as a bar plot depicting the proportion of individual memberships for each cluster: (**a**) K = 2; (**b**) K = 3 (best K). (**c**) Estimated subdivision of the mean membership proportion of each Sicilo-Sarde dairy population in the inferred clusters for K = 3 (best K). SS_B_ = Sicilo-Sarde of Beja region; SS_M_ = Sicilo-Sarde of Mateur region.

To obtain a deeper understanding of the ancestral structure of SS, another analysis was performed without the out-group (Figure 5). Assuming two ancestral clusters (K = 2), all individuals from the two analyzed populations SS_B_ and SS_M_ were split into two clusters (Figure 5a), highlighting the narrative history of the SS resulting from crossbreeding patterns between the Sarda and Comisana Italian dairy breeds. The findings of Tolone et al. [42], showing the subdivision of the group formed by the dairy breeds at K = 3 into (1) Sicilian dairy ancestral cluster including Comisana and (2) Sardinian ancestral cluster presented by Sarda, corroborate our cluster analyze results, highlighting the dual ancestral gene pool in SS sheep resulted from both the Sarda and Comisana gene pools (Figure 5a). Assuming K = 3 (the most likely number of ancestral populations according to the method of Evanno et al. [34], Figure 3b), the two dairy populations tend to be divided into three ancestral gene pools (Figure 5b,c). The proportion of SS_B_ and SS_M_ populations in the three most likely clusters inferred is illustrated in Figure 5c. The proportion of memberships in the different clusters was moderate and showed a slight difference between SS_B_ and SS_M_. In fact, the red cluster contributed 34.5% to SS_B_ gene pool but only 29.6% to the SS_M_ gene pool; the other cluster contributions between the two dairy populations are similar. This distribution highlights the introduction of the Sarda genes in the last decade by artificial insemination, which only affected the SS_B_, thus suggesting the red cluster as being more specific to the gene pool of Sarda sheep. A further analysis including Comisana and Sarda breeds is needed to obtain a better understanding of the SS breed history.

Furthermore, the consideration of three ancestral populations as best assumptions for the SS population structure fits with the history of the sheep introduction from the domestication center in the Fertile Crescent to North Africa and the western Mediterranean basin and following three distinguished waves [45]. The present-day sheep are the miscegenation result of these three ancestral gene pools, as depicted in Figure 5b,c and as revealed by Ben Sassi-Zaidy et al. [19], who studied the genetic structure of Tunisian native meat sheep using microsatellite markers. Ben Jemaa et al. [46], inferring the population structure of some Mediterranean sheep using SNP markers, proved the ancestral relationship between the dairy Sicilo-Sarde breed, and the Italian Sarda and Comisana breeds and revealed the existence of three ancestral gene pool clusters in the genetic structure of the Sicilo-Sarde and other Mediterranean sheep. Furthermore, with SNP markers, Ciani et al. [47] revealed the genetic differentiation of the two insular Sicilian and Sardinian Italian sheep from continental sheep populations, demonstrating the influence of the isolation of these breeds in preserving the ancestral genetic patterns, which reflect the historic maritime waves of sheep introduction to the Mediterranean area. These findings highlight the accuracy and the usefulness of the microsatellites markers in detecting the present-day and historic populations’ genetic patterns, demonstrating the common history of sheep introduction in the western Mediterranean basin before the strong differentiation imposed by selection strength massively applied in the northern Mediterranean rive to obtain modern sheep breeds.

#### 3.2.2. Breed Differentiation and Neighbor Network Analysis

**Figure 6:**
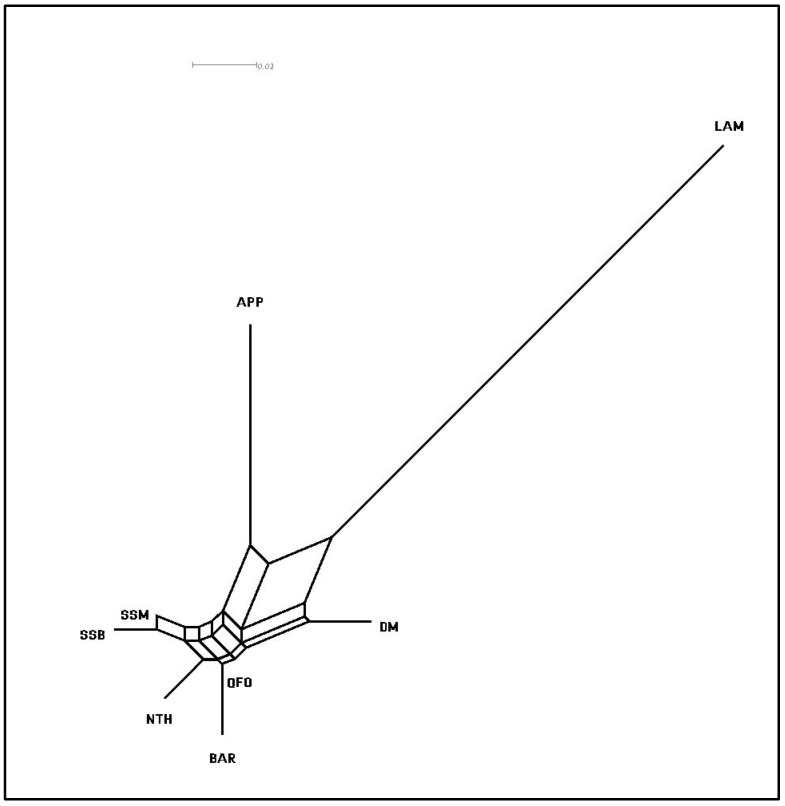
Neighbor network showing the genetic relationship between the unique North African Sicilo-Sarde dairy sheep and the Tunisian and Italian meat sheep breeds based on pair-wise *F*_ST_ distances. SS_B_ = Sicilo-Sarde of Beja; SS_M_ = Sicilo-Sarde of Mateur; Tunisian meat breeds: BAR = Barbarine; QFO = Queue Fine de l’Ouest; NTH = Noire de Thibar; DM = D’man; Italian meat breeds: APP = Appenninica; LAM = Lamon.

The pair-wise *F*_ST_ distance (Table 4) was computed to further highlight the genetic relationships between the SS dairy sheep and the other out-group Mediterranean meat sheep. The lowest *F*_ST_ value (0.001) was detected between SS_B_ and SS_M_, highlighting their genetic closeness. Limited genetic distance was also revealed between the BAR and QFO native Tunisian breeds. NTH breed presented a low differentiation from the group of the SS breed and the group formed by BAR and QFO. The DM originating from Morocco was the most genetically distant from the other North African breeds. The APP and then the LAM Italian breeds were the most genetically differentiated. These results were illustrated in the neighbor network built from the *F*_ST_ distances (Figure 6). The graph clearly separated the dairy SS breed from the other Tunisian meat breeds. The Center Italian APP breed and the Venetian LAM breed clustered into two separate branches. These findings supported the structure analysis depicted in Figure 4.

## 4. Conclusions

In this study, we investigated the genetic variability level, the demographic bottleneck genetic signature and the population structure of the Tunisian Sicilo-Sarde dairy sheep breed using microsatellites analysis. The results confirmed that this endangered breed presented high levels of genetic diversity, no significant inbreeding and a specific genetic structure that could be related to its dairy vocation. This outcome underlined the success of the applied conservation strategy started to save this breed. Further studies need to be carried out to set up an appropriate selection scheme based on the economic potential and the adaptability of this dairy breed.

## Figures and Tables

**Table 1:** Characteristics of the microsatellite loci used to genotype individuals from the Sicilo-Sarde Tunisian sheep dairy breeds. *N*A = number of alleles; AR = allelic richness; PIC = polymorphic information component; *F*_IS_, *F*_IT_ and *F*_ST_ = fixation indices; S.D. = Standard Deviation.

Locus	Chr	Fragment Size (bp)	NA	AR	PIC	*F*_IS_	*F*_IT_	*F*_ST_
Inra023	1	195–221	14	11.52	0.880	0.123	0.119	0.005
Inra063	14	168–206	15	11.18	0.820	0.035	0.043	0.011
OarCP49	17	71–137	16	12.06	0.857	0.019	0.009	−0.010
OarFCB304	19	145–219	15	10.96	0.780	0.008	0.028	0.019
OarFCB20	2	87–117	12	10.05	0.840	−0.056	−0.019	0.036
MAF65	15	119–139	9	7.03	0.791	0.022	0.044	0.024
ILST087	6	142–178	17	13.15	0.897	0.049	0.045	−0.006
OarAE119	19	141–183	11	9.10	0.788	0.182	0.194	0.013
MCM527	5	164–188	11	7.63	0.766	0.183	0.178	0.005
MAF214	16	176–262	5	4.00	0.458	0.170	0.177	−0.005
OarAE129	5	135–163	5	4.90	0.733	0.318	0.294	−0.025
OarCP34	3	101–117	6	5.63	0.782	−0.003	0.018	0.022
OarAE54	25	124–148	12	8.69	0.788	−0.056	−0.029	0.026
TGLA53	12	139–167	14	11.00	0.873	0.183	0.216	0.039
URB058	13	159–211	18	12.23	0.883	0.107	0.127	0.024
CSRD247	14	214–262	15	10.68	0.852	0.288	0.290	0.004
HSC	20	260–296	17	12.48	0.897	0.078	0.081	0.005
Mean			12.47	9.55	0.810	0.100	0.110	0.010
S.D.			4.17	2.81	0.100	0.110	0.100	0.020

**Table 2:** Number of analyzed samples (*N*), mean number of alleles (MNA), allelic richness (AR) obtained with rarefaction method, private allelic richness (*P*_AR_), expected (H_e_) and observed (H_o_) heterozygosity, within-population heterozygote deficiency (*F*_IS_), number of loci deviated from the Hardy–Weinberg equilibrium (E).

Population	N	MNA ± SD	AR	*P*_AR_(25)	H_o_ ± SD	H_e_ ± SD	*F_IS_* ± SD	E
SS_B_	27	10.06 ± 2.54	7.16 ± 1.97	0.69	0.738 ± 0.147 ^b^	0.795 ± 0.096 ^b^	0.090 ± 0.038 ^a^	1
SS_M_	25	9.41 ± 5.03	7.30 ± 1.94	0.68	0.713 ± 0.180 ^a^	0.785 ± 0.112 ^a^	0.121 ± 0.016 ^b^	1

Different superscript letters indicate significant difference (*p* < 0.05).

**Table 3:** Mutation drift equilibrium tests for the tow populations of the Sicilo-Sarde dairy sheep: SS_B_ of Beja region and SS_M_ of Mateur region, performed using Wilcoxon test in BOTTLENECK.

Population	IAM	TPM	SMM
SS_B_	0.00052 *	0.16447	0.99605
SS_M_	0.00871 *	0.66115	0.99451

IAM, infinite alleles model; SMM, stepwise mutation model; TPM, two-phased mutation model; * heterozygosity excess at sign test, *p* value = 5%.

**Table 4:** *F*_ST_ distances (*p* < 0.05) between the breeds analyzed. SS_B_ = Sicilo-Sarde of Beja; SS_M_ = Sicilo-Sarde of Mateur; Tunisian meat breeds: BAR = Barbarine; QFO = Queue Fine de l’Ouest; NTH = Noire de Thibar; DM = D’man; Italian meat breeds: APP = Appenninica; LAM = Lamon.

*F*_ST_ Distances	DM	NTH	QFO	SS_B_	SS_M_	BAR	APP	LAM
DM	-	0.034	0.022	0.046	0.040	0.035	0.073	0.110
NTH		-	0.014	0.025	0.018	0.025	0.069	0.123
QFO			-	0.020	0.016	0.007	0.052	0.110
SSB				-	0.001	0.026	0.071	0.138
SSM					-	0.026	0.051	0.117
BAR						-	0.068	0.132
APP							-	0.135
LAM								-

## Data Availability

The data presented in this study are available within the article.
